# Does Drug-Eluting Bead Transcatheter Arterial Chemoembolization Improve the Management of Patients with Hepatocellular Carcinoma? A Meta-Analysis

**DOI:** 10.1371/journal.pone.0102686

**Published:** 2014-08-01

**Authors:** Shilong Han, Xiaoping Zhang, Liling Zou, Chenhui Lu, Jun Zhang, Jue Li, Maoquan Li

**Affiliations:** 1 Department of Interventional & Vascular Surgery, Shanghai Tenth People's Hospital, Tongji University School of Medicine, Shanghai, China; 2 Heart, Lung and Blood Vessel Center, Tongji University School of Medicine, Shanghai, China; St. Luc University Hospital, Belgium

## Abstract

**Background:**

Drug eluting beads (DEB) are relatively new embolic agents that allow sustained release of chemotherapeutic agents in a localized fashion to the tumor. This technique is associated with reduced systemic side effects relative to systemic chemotherapy and an increase in the dose of antineoplastic agent delivered to the lesion. The meta-analysis was undertaken to assess the effectiveness of DEB-transcatheter arterial chemoembolization (TACE) in the management of hepatocellular cancer.

**Methods:**

We searched the Web of Science, PubMed, EBSCO, EMBASE, the Wiley Library and Google Scholar for studies on DEB-TACE in the management of hepatocellular cancer from 1979 to April 2013. The risk of bias was assessed using RevMan 5·1. Random and fixed-effects meta-analytical models were used where indicated, and between-study heterogeneity was assessed. Disease control, complications and severe complications were recorded.

**Results:**

Five studies met the selection criteria, three RCTs and two case-control studies, published from 2010 to 2012, included 217 patients in the DEB-TACE group and 237 in the conventional-TACE group. There was no significance over disease control (OR 2.27, 95% CI 0.78–6.63) with moderate between-study heterogeneity (χ^2^ = 6.83, degrees of freedom [df] = 3; p<0.08; I^2^ = 56%). Complications in both groups were assessed and no significant difference was observed (χ^2^ = 6.34, degrees of freedom [df] = 4; p<0.18; I^2^ = 37%). Severe complications were also assessed and no significant difference was observed (χ^2^ = 6.47, degrees of freedom [df] = 4; p<0.17; I^2^ = 38%). No publication bias relating to the above outcomes was detected by funnel plot. DEB-TACE benefited disease control without an increase in complications and severe complications.

## Introduction

Hepatocellular carcinoma (HCC) is the fifth most common malignancy world-wide, with half a million new cases reported every year. HBV, HCV and alcoholic liver disease are the major risk factors in the etiology of HCC, and Southeast Asia and sub-Saharan Africa are the most affected regions. Over the past two decades, the incidence rate of HCC has tripled and the 5-year survival rate has increased from more than 1% to nearly 12% in the USA [1]–[3]. Although the early detection of small HCC has received much attention through recommended surveillance strategies in order to obtain a better response to curative treatments such as liver resection, liver transplantation and locoregional procedures, most patients with HCC are not candidates for curative therapies even at time of diagnosis due to poor liver function or tumor characteristics such as large or multifocal lesions. Survival rates for intermediate stage patients at 1, 3 and 5-years are 80%, 65% and 50%, and for patients with advanced disease are 29%, 16% and 8%, respectively [4].

Since the first report on transcatheter arterial chemoembolization (TACE) in the 1970s, TACE using Lipiodol mixed with antineoplastic emulsion and gelatin particles, now referred to as conventional TACE (cTACE), has been widely performed for unresectable HCC and other cancers as a palliative therapy [5]. Although the effectiveness of this technique remains controversial, a meta-analysis which included five randomized controlled trials (RCT) showed that TACE significantly reduced the overall 2-year mortality rate compared with nonactive treatment [6]. The drug eluting bead (DEB) is a new embolic agent as well as an antineoplastic agent carrier [7]. The use of DEBs which release the antineoplastic gent in the lesion in a controlled fashion is a new technique and has been shown to be associated with a reduction in systemic side effects and an increase in dose of the antineoplastic agent in the local lesion [8]–[16]. Pooled data from six clinical trials showed high local response rates ranging from 52% to 81% [17]–[24]. However, there is no strong evidence to show whether the cTACE is better than DEB-TACE in any terms of effectiveness. We performed a meta-analysis to determine whether DEB-TACE was more effective than cTACE in patients with HCC.

## Method

### Search

A computerized bibliographic search from 1979 to April 2013 was conducted on Web of Science (including MEDLINE), PubMed, EBSCO, EMBASE, the Wiley Library and Google Scholar, using the following terms: DEB, drug eluting bead, drug eluting microsphere and TACE. The related-articles function was allowed to expand the search findings and all abstracts, studies and citations were reviewed irrespective of language. We also searched the related study on www.clinicaltrials.gov in an attempt to find unpublished studies. The updated search date was April 1^st^, 2013.

### Study selection

Studies were excluded by reading the titles or abstracts, and all studies based on animal or in vitro experiments, single-arm studies, case reports, reviews and studies on metastatic liver lesions were excluded. Following careful reading of abstracts and full-texts, studies which examined DEB-TACE versus cTACE were included if the study complied with the following requirements: clinically diagnosed HCC, Child-Pugh A or B, case-controlled trial or RCT outcomes assessed by modified Response Evaluation Criteria in Solid Tumor (RECIST) or the Europe Association for the Study of Liver Disease (EASL) measurement, and complications were recorded. For repeat studies conducted by the same author, the most recent and informative study was adopted if there were overlapping data.

### Assessment of risk of bias in eligible studies

Two review authors (SH, LZ) independently assessed the risk of bias in each included study using Revman 5·1. Agreements were reached by discussion between the two review authors if there were disagreements on specific items in the studies.

### Data extraction

All the articles searched were managed by Endnote X5. One reviewer (SH) retrieved articles that potentially met the inclusion criteria. The full text was requested from the authors if the study was included but not readily available on the database. From each included study, we extracted data on patient characteristics, demographics, country, study design, sample size, etiology of HCC, Child-Pugh score, Barcelona Clinic Liver Cancer (BCLC) classification, antineoplastic dose, DEB dose, tumor response, complications and severe complications from both the DEB-TACE group and the cTACE group. The primary endpoint of this meta-analysis was tumor response assessed by the EASL criteria or modified RECIST which focused on viable tumor on imaging evidence and included partial response (PR), complete response (CR), stable disease (SD), and progressive disease (PD) [25]. For converting continuous to dichotomous, we reclassified the PR, CR and SD in raw data as disease control (DC), which means disappearance of all detectable tumor, or a decrease of more than 50% or 30%, or an increase of <20% or 25%, without the appearance of new lesions based on CT or MRI The minimum medical imaging follow-up time was 4 weeks after the procedure in all the included studies. Complications, namely post-TACE complications, were acute liver impairment, encephalopathy, ascites, epigastric pain and fever. Severe complications included increased hospital stay, injury or death.

### Statistical analysis

This meta-analysis was carried out in compliance with the recommendations of the Cochrane Reviewers' handbook 4.2.2 [26]. The odds ratio (OR) was used as the summary statistic for statistical analysis of dichotomous variables and represented the odds of a favorable event occurring in the DEB-TACE group compared to the cTACE group and indicated the relative risk. An OR of more than 1 favored the DEB-TACE group. The point estimate of the OR was considered to be statistically significant or a p-value <0.05 was significant if the 95% CI did not include the value 1.

A fixed-effect or a random-effect model was used for the meta-analysis where indicated. Heterogeneity between the groups was evaluated by the χ^2^ and I^2^ statistic, and higher χ^2^ and I^2^ statistic values indicated greater heterogeneity between the groups [27]. The hypothesis of homogeneity between the groups was thought to be invalid if the p-value was <0.1 and the random-effect model was adopted and the cause of heterogeneity was investigated. The fixed-effect model was then considered. Publication bias was analyzed using a funnel plot. A two-tailed p-value <0.05 was considered statistically significant. Analysis was performed using Review Manager version 5.1.5 for Windows.

## Results

**Figure 1:**
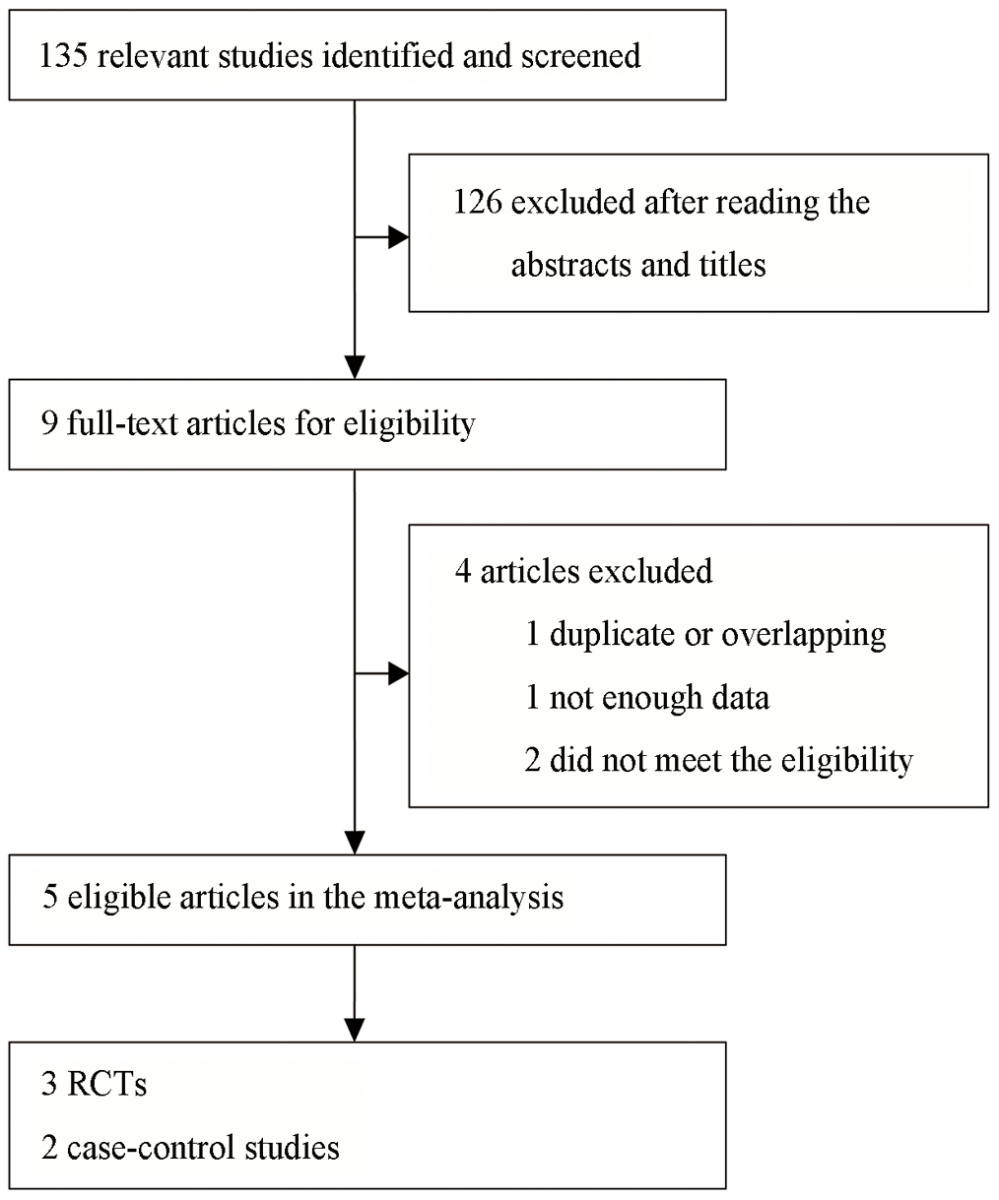
Search strategy.

In total, 135 potentially relevant articles were identified using the search strategy (Figure 1). One hundred and six articles were excluded after reading the title and abstract, which included 27 animal or in vitro experiments, 48 single arm studies, 18 studies involved patients with liver metastasis, 18 review articles, nine comparative studies which either had no full texts or were ineligible, four case reports and two official files. Following a further review of the remaining nine articles, four were excluded due to overlapping data, not enough information or were ineligible, of which Frenette, who conducted an article involving 274 patients, provided the baseline characteristics of patients and are working on the outcomes.

Therefore five studies published between 2010 and 2012 met the selection criteria and were included in this meta-analysis [23], [ 28]–[31]. The references in these studies did not provide further studies for review. The analysis was performed on 217 patients in the DEB-TACE group and 237 in the cTACE group. Of these five studies, three were RCTs and two were case-control studies, of which four were from Europe and one from Asia.

**Figure 2:**
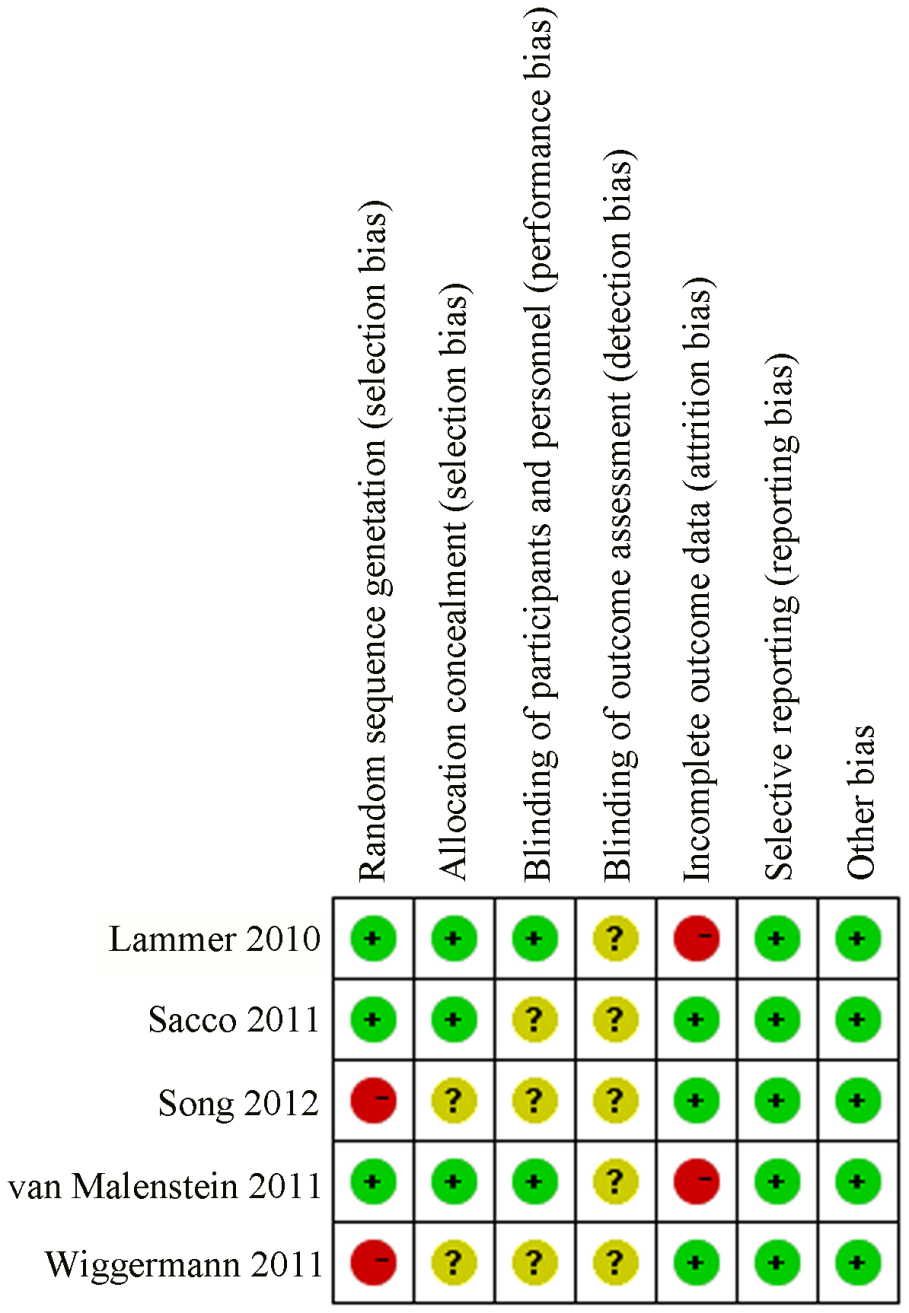
Risk of bias summary: review of authors' judgments on the risk of bias of each item in each included study. All the blinding method of these articles are unknown but reported the low selective reporting and other bias, which prove the reliability of these studies.

**Figure 3:**
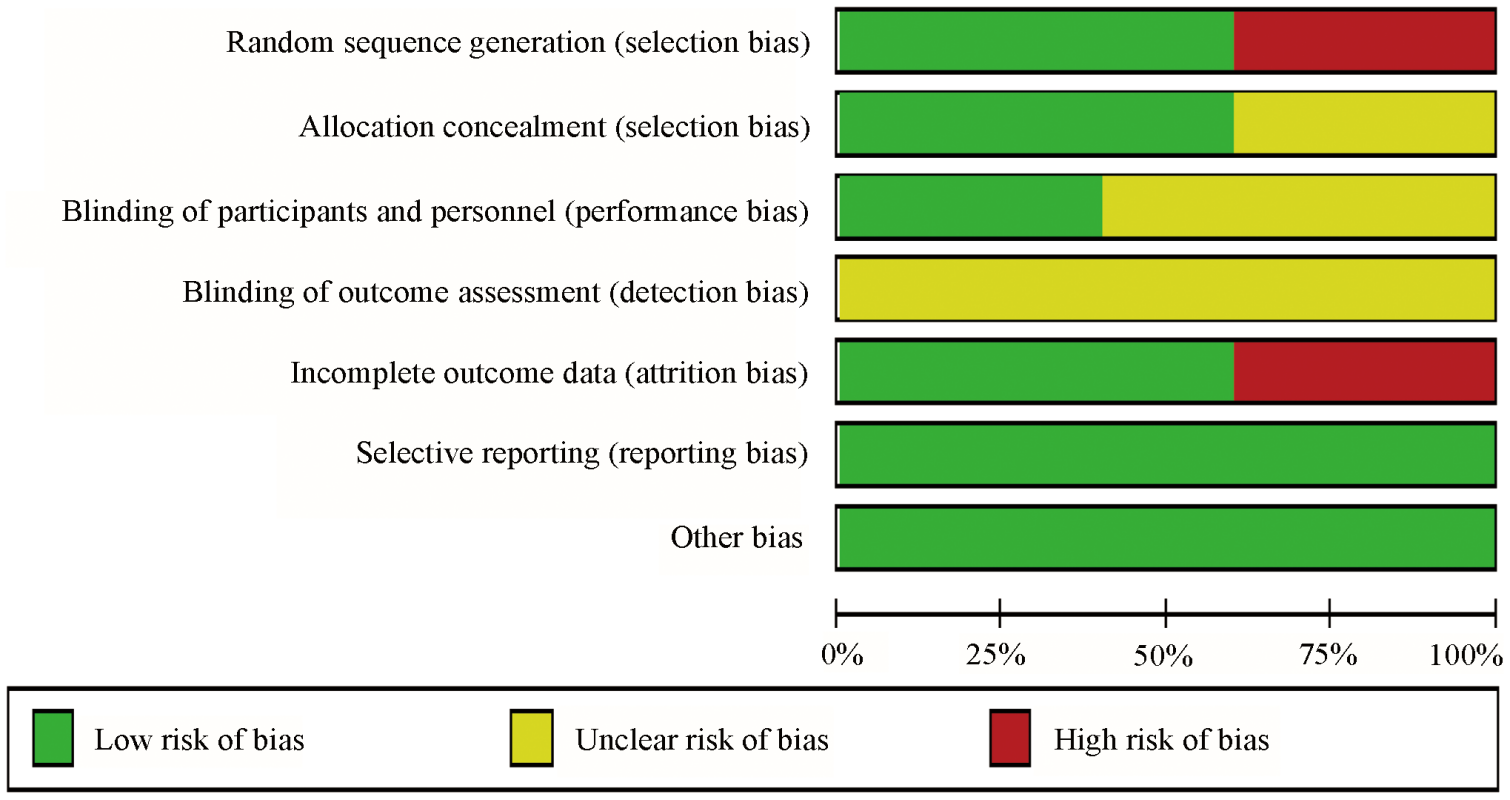
Risk of bias graph: review of authors' judgments on the risk of bias in each item presented as percentages in all included studies, as described in Figure 2.

All three RCTs were considered to be of high methodological quality. One of the RCTs conducted by Lammer and colleagues was an international, multicenter, prospective and single-blind study which included 189 patients and constituted the major part (41.6%) of this meta-analysis. The other two RCTs were single center and randomized studies, but failed to provide the specific randomization method. Two case-control studies focused on a definite issue with appropriate method, but failed to provide the exposure factor. Song and colleagues concluded that DEB-TACE resulted in a better treatment response than cTACE, while Wiggermann concluded that there was no significant increase in terms of disease control between the treatment groups. The risk of bias for each study is shown in Figure 2 and Figure 3. The study characteristics and patient demographics are shown in Table 1. Two studies described treatment cycles patients received. In Lammer's study, 82% patients received two cycles of treatment in each group, and 61%, 57% patients received three cycles in DEB-TACE and cTACE group, respectively. Whereas, in Sacco's study, 22.4% received two cycles of treatment in each group and 4.5%, 5.5% received three cycles in DEB-TACE and cTACE group. However, no significant difference was found in both studies.

**Table 1:** Demographic characteristics of the included studies.

	Year	Article Type	Bead Type	Patient	Mean age	Etiology	Child-Pugh	BCLC	Drop out	Median Follow-up
				D/C	D/C	HCV/HBV/AC/O	A/B	A/B/C		
**Lammer**	2010	RCT	DC Bead	93/108	67·3/67·4	40/34/100/46	166/35	53/148/0	12	6mo
**Sacco**	2011	RCT	DC Bead	33/34	71·3/68·7	47/8/0/12	54/13	44/23/0	0	1mo
**Song**	2012	Retrospective	DC Bead	60/69	61·7/59·4	16/90/16/7	119/10	55/74/0	0	18mo
**van Malenstein**	2011	RCT	SAP	16/14	67·3/56·6	8/4/14/4	28/2	3/19/8	5	6w
**Wiggermann**	2011	Retrospective	DC Bead	22/22	70·3/67·7	9/0/9/26	44/0	5/32/5	0	8mo

Child-Pugh = Child-Pugh classification. BCLC = Barcelona Clinic Liver Cancer tumor staging. D = DEB-TACE group. C = cTACE group. O = others. RCT = randomized controlled trial.

**Figure 4:**
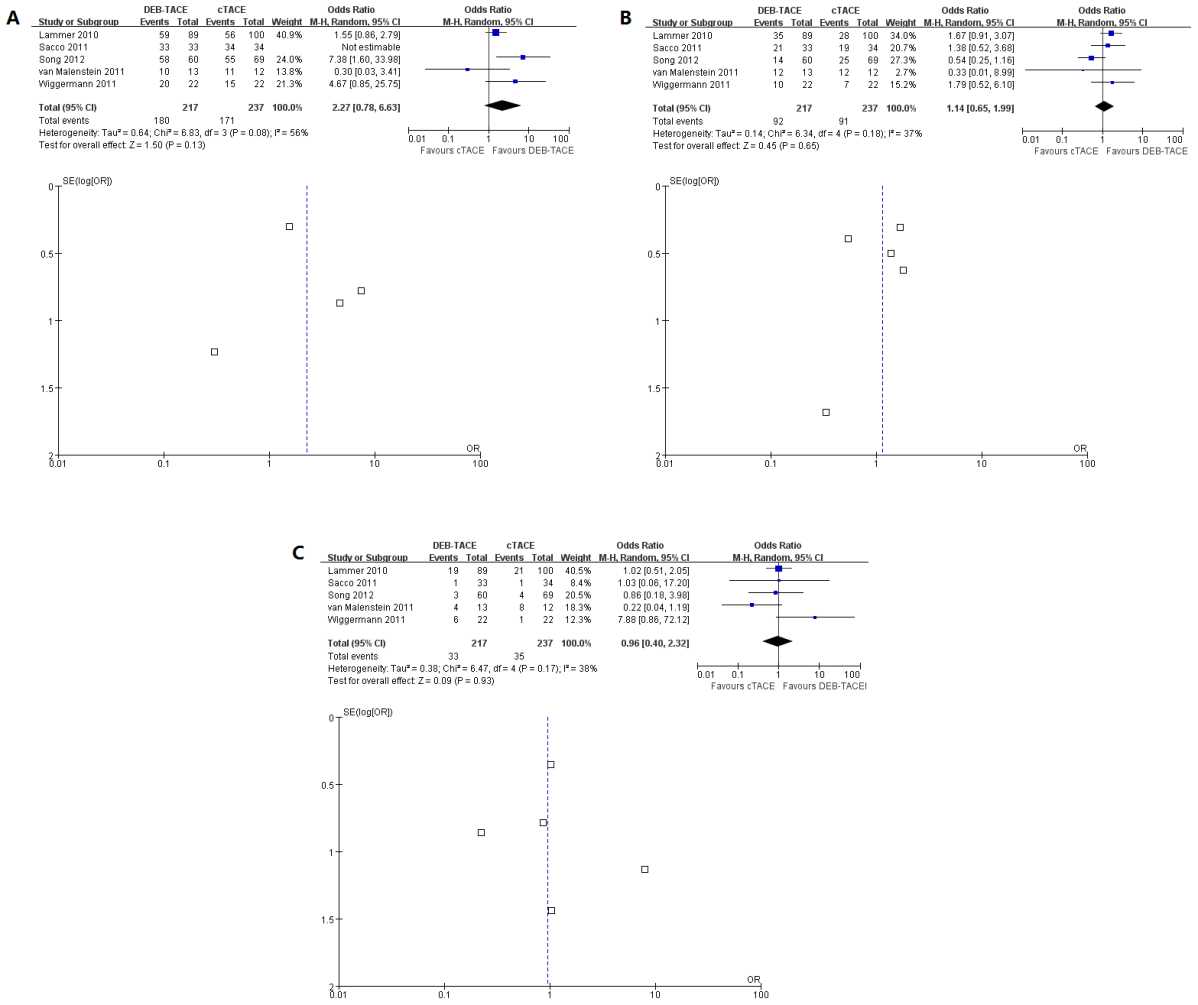
A. Forest plot comparison of DEB-TACE vs. cTACE group in terms of disease control shows no significance of odds ratio. The Lammer's study takes greater weight against other studies. No publication bias in the funnel plot. B. Forest plot comparison of DEB-TACE vs. cTACE group in terms of complications show there is no significance of odds ratio and no publication bias. C. Forest plot comparison of severe complications show there is no significance of odds ratio between these two groups and report no publication bias.

Pooled data from the included studies which assessed disease control showed no significance between two groups (OR 2.27, 95% CI 0.78–6.63) with moderate between-study heterogeneity (χ^2^ = 6.83, degrees of freedom [df] = 3; p<0.08; I^2^ = 56%). No publication bias was detected by the funnel plot (Figure 4A).

Data on complications in both groups were assessed and no significant difference between the two groups was observed (χ^2^ = 6.34, degrees of freedom [df] = 4; p<0.18; I^2^ = 37%). No publication bias was detected by the funnel plot (Figure 4B).

Data on severe complications in the studies were assessed and no significant difference between the two groups was observed (χ^2^ = 6.47, degrees of freedom [df] = 4; p<0.17; I^2^ = 38%). No publication bias was detected by the funnel plot (Figure 4C).

## Discussion

This meta-analysis examined the efficacy of DEB-TACE versus cTACE in patients with HCC. Though the analytical results showed no statistical significance, but the odds ratio is high in terms of disease control and there is no increase in complications or severe complications. There are currently two types of DEB on the market: polyvinyl alcohol-based microspheres (DC Bead, BioCompatibles Ltd., Farnham, UK) and superabsorbent polymer microspheres (HepaSphere, Biosphere Medical, Rockland, MA, USA) [22]. The initial report on the DEB was by Lewis and colleagues in 2006 which presented detailed in vitro characterization of the DEB (DC Bead) [7]. The study showed that modeling of the kinetics of drug elution from the beads in vitro at a loading dose of 25 mg/ml yielded calculated half-lives of 150 hours for the 100–300 µm size range to a maximum of 1,730 hours for the 700–900 µm size range, which was dependent on the ionic strength of the elution medium in comparison with an unstable Lipiodol emulsion that showed a rapid loss of drug with a half-life of approximately 1 hour. Therefore, long-term interaction with the tumor and the high concentration of antineoplastic agent in the lesion were the main advantages over the conventional drug carrier and embolic agent. The largest study as well as RCT, conducted by Lammer, did not show significance in terms of disease control, but the subgroup analyses showed that in 67% of patients with more advanced disease (Child-pugh B, ECOG 1, bilobar or recurrent disease), the incidence of overall survival and disease control were statistically higher (p = 0.038 and p = 0.026, respectively) in DEB-TACE group compared with the cTACE group. The supplementary post hoc analysis indicated that the incidence of severe adverse events within 30 days of a procedure was consistently lower, and AST as well as ALT was significantly less in the DEB-TACE group. The largest retrospective study from Song showed the treatment response in the DEB-TACE group was significantly higher than that in the cTACE group (p<0.001) and the subgroup analysis according to BCLC stage indicated that the treatment responses in intermediate stage was significantly better in DEB-TACE group than cTACE group as well (p<0.001). We may therefore surmise that DEB-TACE could improve the clinical effectiveness in patients with more advanced HCC.

## Limitations

There are limitations in this meta-analysis. First, there are limited numbers of articles focusing on DEB-TACE, and even less on the comparison of cTACE and DEB-TACE. The ongoing clinical trials on DEB-TACE are mostly single-arm study. Second, only five studies which included 454 patients were eligible for the inclusion criteria, and four of the included studies have small sample sizes. Therefore, a sensitivity analysis to gain further knowledge on the factors affecting tumor response and other outcomes was not applied. Third, cTACE performed in different medical centers can vary either due to the different embolic agents used or different levels of manual expertise. In terms of the antineoplastics used, four studies used adriamycin and one used cisplatin in the cTACE group. Forth, medical imaging follow-up varied greatly from 6 weeks to 18 months and is quite short, which may have affected the primary endpoint of the meta-analysis. Fifth, survival time was not described in the meta-analysis although three studies recorded survival time in different ways. Finally, we noted that the primary endpoint of the largest RCT by Lammer and colleagues played an important role in this meta-analysis (weighted, 40.9%) and the initial in vitro study was sponsored by DC Bead, BioCompatibles Ltd.

A recent systematic review which focused on the safety and efficacy of DEB-TACE in HCC patients showed that there was sufficient evidence to support the use of the DC Bead as a safe and effective embolic treatment, however, there was overlapping information in this meta-analysis [5]. In our meta-analysis, no severe adverse events related to the safety of DEB were reported. Complications and severe complications between the two groups were not significantly different. With the exception of the RCTs and case-control studies, almost 40 single-arm studies included information on the efficacy of DEB since 2006 which showed the promising application of this treatment in clinical practice [32]–[55]. Based on this meta-analysis, further high quality randomized clinical trials with longer follow-up may focus on patients with more advanced stage, who may be of more benefit with DEB-TACE.

## Supporting Information

Checklist S1 **PRISMA Checklist.**
(DOC)
